# Three-Dimensional Analysis of the Contact Pattern between the Cortical Bone and Femoral Prosthesis after Cementless Total Hip Arthroplasty

**DOI:** 10.1155/2016/8052380

**Published:** 2016-01-10

**Authors:** Hiroshi Wada, Hajime Mishima, Hisashi Sugaya, Tomofumi Nishino, Masashi Yamazaki

**Affiliations:** ^1^Department of Orthopaedic Surgery, Graduate School of Comprehensive Human Sciences, University of Tsukuba, 1-1-1 Tennodai, Tsukuba, Ibaraki 305-8575, Japan; ^2^Department of Orthopaedic Surgery, Institute of Clinical Medicine and University Hospital, University of Tsukuba, 1-1-1 Tennodai, Tsukuba, Ibaraki 305-8575, Japan; ^3^Division of Regenerative Medicine for Musculoskeletal System, Department of Orthopaedic Surgery, Faculty of Medicine, University of Tsukuba, 1-1-1 Tennodai, Tsukuba, Ibaraki 305-8575, Japan

## Abstract

The cementless stem Excia (B. Braun, Melsungen, Germany) implant has a rectangular cross-sectional shape with back-and-forth flanges and a plasma-sprayed, dicalcium phosphate dihydrate coating from the middle to proximal portion to increase initial fixation and early bone formation. Here, the conformity of the Excia stem to the femoral canal morphology was three-dimensionally assessed using computed tomography. Forty-three patients (45 hips) were examined after primary total hip arthroplasty with a mean follow-up of 27 ± 3 months (range: 24–36 months). Spot welds occurred at zone 2 in 16 hips and at zone 6 in 24 hips, with 83% (20/24 hips) of those occurring within 3 months after surgery. First- (*n* = 12 hips), second- (*n* = 32), and third- (*n* = 1) degree stress shielding were observed. The stem was typically in contact with the cortical bone in the anterolateral mid-portion (100%) and posteromedial distal portions (85%). Stress shielding did not progress, even in cases where the stems were in contact with the distal portions. The anterior flange was in contact with the bone in all cases. The stability of the mid-lateral portion with the dicalcium phosphate dihydrate coating and the anterior flange may have inhibited the progression of stress shielding beyond the second degree.

## 1. Introduction

**Figure 1:**
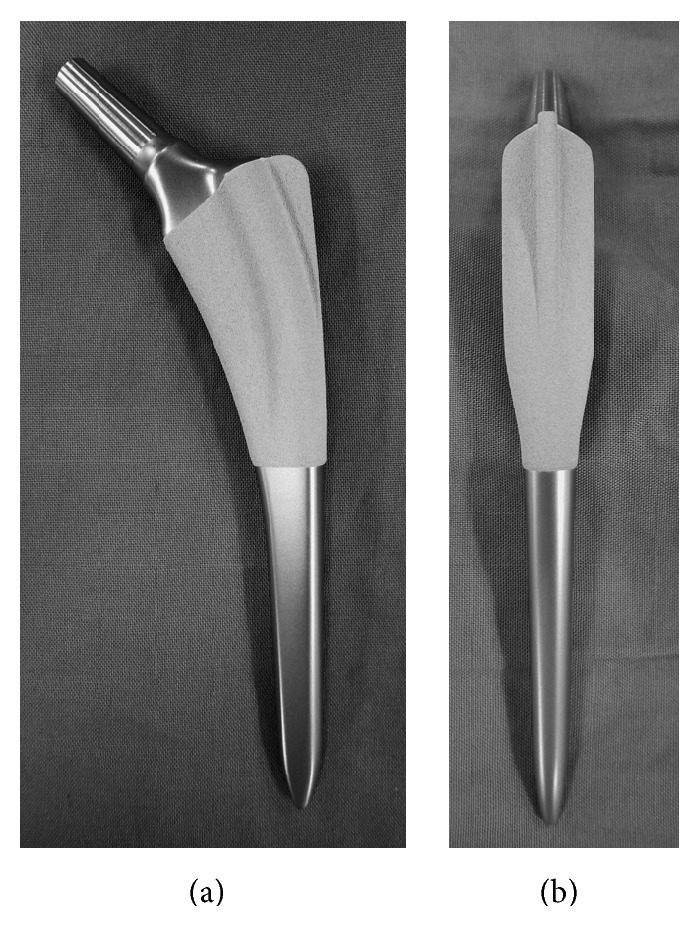
Anterior (a) and lateral (b) views of the cementless stem Excia.

In cementless total hip arthroplasty (THA), the distal fixation between the femoral stem and the cortical bone may lead to distal load transfer and proximal stress shielding [1]. The cementless stem Excia (B. Braun, Melsungen, Germany) implant has a rectangular cross-sectional shape with back-and-forth flanges. The thin lateral trochanter wing preserves the cancellous bone of the major trochanter while facilitating rotational stability. After fixation through excavation of the cortical bone of the metaphysis, rotational stability and proximal load transfer are expected. Good clinical results have been reported with the Excia stem [2]. The Excia stem has a rough titanium, plasma-sprayed, dicalcium phosphate dihydrate (DCPD) coating layer in its central and proximal portions to increase early bone formation by releasing calcium and phosphate ions (Figure 1) [3]. Proximal load transfer is expected to occur through the promotion of bone apposition in the metaphysis. However, the DCPD coating may result in early loosening of the implant because of soft tissue reactions and osteolysis [4]. To the best of our knowledge, no previous studies have estimated the contact between this specific Excia stem and the femoral canal.

Thus, the goal of the present study was to determine the conformity and the initial fixation of the Excia stem with the femoral canal morphology by three-dimensional computed tomography (CT) analysis. The first aim was to show the stability of the Excia stem during the early postoperative phase. The second aim was to assess the contact pattern between this uniquely shaped stem and the cortical bone, as well as clarify the effects of bone remodeling around the stem.

## 2. Materials and Methods

This study was performed in accordance with the ethical standards of the 1964 Declaration of Helsinki as revised in 2000 and received approval from our institutional review board committee. All patients provided written informed consent. The subjects comprised 43 patients (45 hips) who underwent unilateral or bilateral primary THA with the Excia stem at our facility from 2009 to 2010. In all patients, a Plasmacup SC acetabular component (B. Braun, Melsungen, Germany) was implanted with third-generation ceramic-on-ceramic bearing couples. The right hip was affected in 31 cases, and the left hip was affected in 14 cases. The main indication for THA was secondary osteoarthritis caused by acetabular dysplasia in 34 patients (79%), osteonecrosis in 7 patients (16%), and rheumatoid arthritis in 2 patients (5%). The patients were 8 men and 35 women. Their mean age was 65 ± 11 years (range: 35–85 years), mean BMI was 24.3 ± 3.8 kg/m^2^ (range: 17.0–33.3 kg/m^2^), and mean follow-up was 27 ± 3 months (range: 24–36 months). All patients completed the follow-up appointments. The preoperative femur morphology was examined using the canal flare index (CFI) [5]. The mean CFI was 4.0 ± 0.6 (range: 2.9–5.9), with 39 hips having normal canals, 5 hips having champagne-fluted canals, and one hip having a stove-pipe canal. All surgeries were performed using minimally invasive surgical techniques. Full weight bearing was permitted as soon as pain diminished.

### 2.1. Clinical Evaluation

We assessed the patients using the Japanese Orthopaedic Association evaluation standard for hip joint function (JOA score) and the Harris Hip Score (HHS) preoperatively and at the final follow-up appointment. Any perioperative complications and thigh pain were also assessed.

### 2.2. Radiological Evaluation

Standardized anteroposterior and axial radiographs of the hips were taken at 1 and 2 weeks and 1, 2, 3, and 6 months postoperatively, as well as every 6 months thereafter. The biological fixation of the stem was assessed on the radiographs obtained at the final follow-up observation using Engh's criteria [6]. Stem subsidence and bone remodeling around the stem were recorded at each assessment point. The conventional Gruen zones [7] were used to assess bone remodeling in the postoperative radiographs. Cortical hypertrophy, spot welds, reactive lines, and stress shielding were recorded. Stress shielding was classified from 0 (none) to 4 (fourth degree) based on Engh's classification [1].

**Figure 2:**
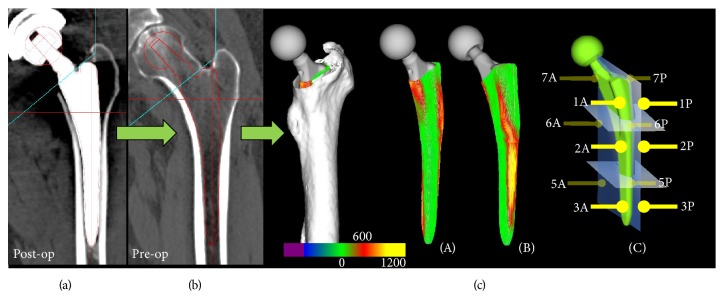
Virtual implant model generated from CAD data (a) and aligned and fitted to the contour of the real prosthetic stem postoperatively. The virtual model was aligned in the same position with a preoperative CT image (b). The contact pattern images were three-dimensionally visualized using a color map based on the CT values around the stem (c). The stem was defined as being in contact with the cortical bone if the CT value around the stem was ≥600 HU. Red color indicates 600 HU and yellow color indicates 1200 HU. (A) Anteromedial view. (B) Anterolateral view. (C) Modified Gruen zone classification; for example, zone 2A/zone 2P indicates the anterior/posterior portion of zone 2.

The pattern of contact between the stem and the cortical bone was assessed using CT images obtained from the patients before and at 1 week after the operation. We analyzed the CT images of 1 mm thick slices obtained from a total of 34 hips; one hip from a patient aged under 40 years and 10 initial hips were excluded from the analysis because CT images were not obtained. The neck-shaft angle and neck anteversion were measured using the CT images taken before surgery. It was impossible to detect the cortical bone around the stem because of the reaming procedure used and the implant halation on the CT images taken after surgery. Therefore, a virtual implant model was aligned on the CT image taken before surgery as a reference for the same position shown on the CT image taken after surgery. This virtual implant model generated from the computer-aided design (CAD) data was aligned and fitted to the contour of the real prosthetic stem after surgery using the digital template software provided for a THA implant (ZedHip; LEXI, Tokyo, Japan). This software allowing reconstruction of a three-dimensional model was described previously [8]. The virtual implant model was manually aligned on the CT image taken before surgery as a reference to the same position shown on the CT image taken after surgery. All of the CT values around the virtual stem model were three-dimensionally visualized on the CT images before surgery using an automated program. The thickness of the surface coating in the proximal portion was determined using the stem CAD data. Aamodt et al. [9] reported that the CT value of the bone at the corticocancellous interface was 600 Hounsfield units (HU) in human cadaveric femora. They rasped the proximal femoral canal and cut it into several sections corresponding to the CT image slices. We defined contact between the stem and the bone as CT values greater than 600 HU around the stem on the CT images before surgery (Figure 2). We believe that three-dimensional evaluation was necessary to accurately assess the contact pattern and determine the modified Gruen zone classification, in which each zone was divided into anterior and posterior portions (Figure 2(C)).

### 2.3. Statistical Analysis

JMP 10.0.4 software (SAS Institute Inc., Cary, NC, USA) was used for all statistical calculations. The JOA score and HHS before and after surgery were compared using the Wilcoxon signed-ranks test. The hips were categorized into two groups: first-degree stress shielding group and second- or greater-degree stress shielding group. Differences between the groups were evaluated using Student's *t*-test for stem size, height, BMI, and CFI, Welch's *t*-test for age, the chi-square test of independence for spot welds, and Fisher's exact probability test for reactive lines. Furthermore, the relationships of the portions in contact with the bone were evaluated using the chi-square test of independence for stress shielding and reactive lines, Kruskal-Wallis test for stem size, and multiple regression analysis using stepwise methods for the CFI, neck-shaft angle, and neck anteversion. *P* values less than 0.05 were considered statistically significant.

## 3. Results

### 3.1. Clinical Evaluation

No patients had thigh pain at the final follow-up. The mean JOA score improved significantly from 51.7 ± 12.7 (range: 27–83) points preoperatively to 90.4 ± 8.2 (range: 60–99) points at the final follow-up. The mean HHS improved from 54.5 ± 13.8 (range: 22–85) points preoperatively to 91.3 ± 8.1 (range: 64.4–100) points at the final follow-up. One fissure found in the proximal femur in one hip was not displaced, and it healed uneventfully.

### 3.2. Radiological Evaluation

The biological fixation of the stem was stable in all cases, with bone ingrowth found in 43 hips and fibrous ingrowth in 2 hips. Stem subsidence was observed in 3 hips within 1 month of surgery but did not progress.

**Figure 3:**
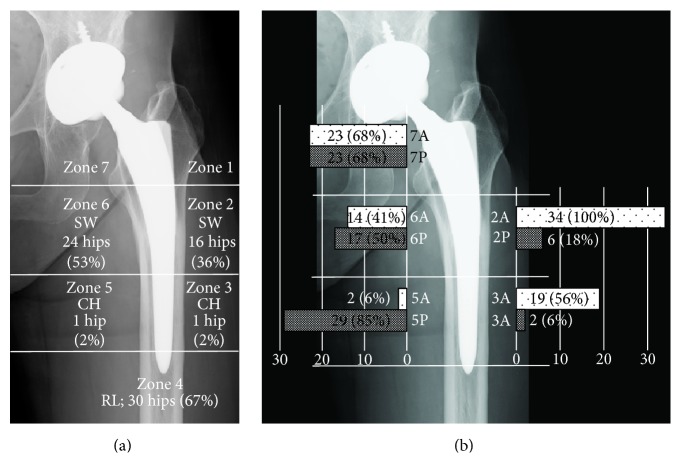
Bone remodeling around the stems (a). SW: spot welds; CH: cortical hypertrophy; RL: reactive lines. (b) Numbers indicate the numbers of hips in which the stem was in contact with the cortical bone using the modified Gruen zone classification.

**Figure 4:**
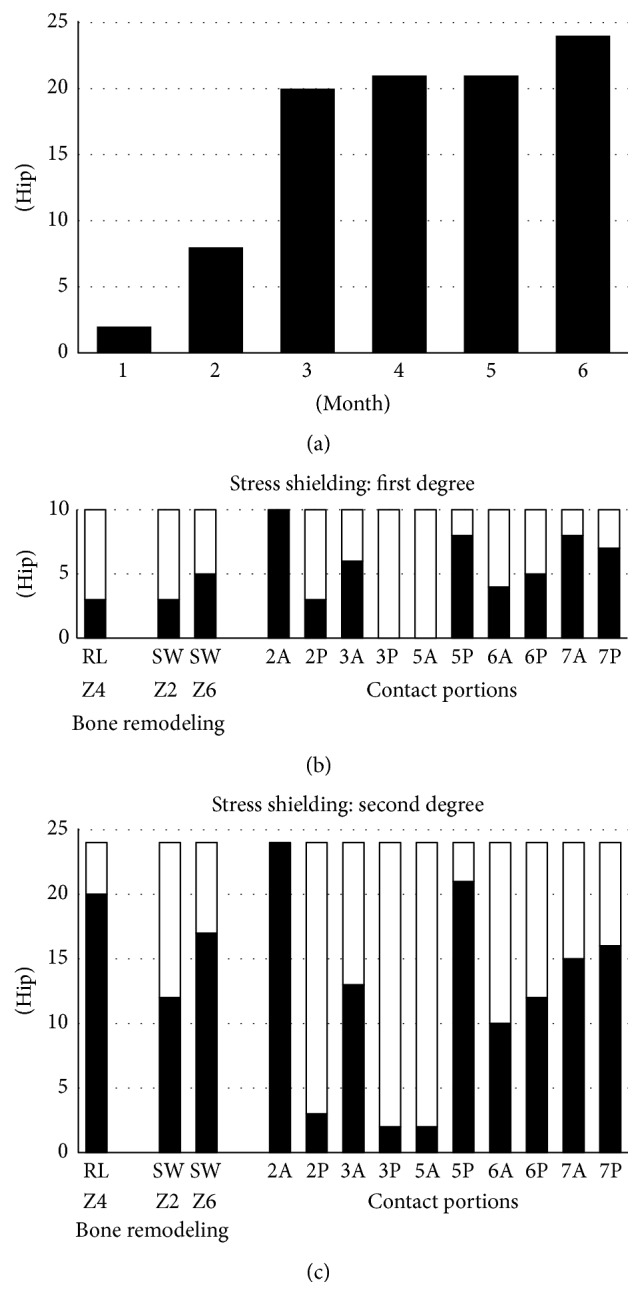
Cumulative frequency of the appearance of spot welds over time (a). Stress shielding of the first (b) and second or greater (c) degree is shown. Solid bars indicate the numbers of hips showing bone remodeling (left) and contact with the bone by portion (right), while the open bars indicate the number of hips without bone remodeling (left) and bone contact (right).

Cortical hypertrophy was found at zone 3 in one hip and zone 4 in one hip. Reactive lines around the tip of the stem (zone 4) were recorded in 30 hips. Spot welds occurred at zone 2 in 16 hips and zone 6 in 24 hips, and all of these appeared at the distal end of the DCPD coating (Figure 3(a)). Furthermore, spot welds were found within 3 months after surgery in 83% (20/24) of the hips in which spot welds were observed (Figure 4(a)). First- (*n* = 12 hips), second- (*n* = 32), and third- (*n* = 1) degree stress shielding were observed. No hips with fourth-degree stress shielding were observed. Counting only the 34 patients whose CT data were obtained, the mean CFI was 3.9 ± 0.6 (range: 3.2–5.1), with 31 hips having normal canals and 3 hips having champagne-fluted canals. The stem was typically in contact with the cortical bone at zone 2A (100%) and zone 5P (85%) (Figure 3(b)). The mean neck-shaft angle was 130.4 ± 6.2 (range: 119–146) degrees and the mean neck anteversion was 39.2 ± 13.4 (range: 15–72) degrees. The hips were categorized into two groups according to the stress shielding grade. The appearance ratio of bone remodeling and the portions in contact with the bone are shown in Figure 4 ((b) first degree; (c) second degree or greater).

### 3.3. Quantitative Comparisons

No significant differences in stem size, height, BMI, CFI, age, or spot welds were found between the first-degree and second- or greater-degree stress shielding groups. However, stress shielding was significantly higher in hips that had reactive lines in zone 4. No significant differences in stress shielding, reactive lines, spot welds, or stem size were found based on the portion in contact with the bone. Similarly, the regressions of the CFI, neck-shaft angle, and neck anteversion with the portion in contact with the bone were not significant.

## 4. Discussion

In the present study, no loss of stability was found during the early postoperative period, and spot welds formed rapidly at zones 2 and 6. Tricalcium phosphate and hydroxyapatite (HA), as well as DCPD, are dissolved during the amorphous period, but HA remains at the interface even after osteointegration [10]. Decreases in the calcium-to-phosphate molar ratio increase the dissolution rate. Increases in the release of Ca^2+^ and PO_4_^3−^ ions can promote the adhesion of many growth factors and proteins to the surface of the implant, as well as the activity and gene expression of osteoblasts [3, 4, 11]. The DCPD coating is believed to act as a heterogeneous center for HA growth during early bone formation and is mostly dissolved after 1 week [11]. However, this highly soluble DCPD coating does not compromise the mechanical stability of the bone-implant interface during the early stage. A thin layer of DCPD can be applied by electrochemical cathodic deposition to a porous substrate without reducing porosity or interconnectivity.

Bone remodeling was previously observed as early as 4 and 8 weeks, with Haversian canals identified at 12 weeks in a sheep model [4, 11]. In the present study, the Excia stem's DCPD coating was applied by the same methods, and spot welds were found within 3 months after surgery in 83% of the hips that developed spot welds (Figure 4(a)). Spot welds may contribute to implant stability during the early phases after surgery because they were observed as a result of cancellous bone formation. Saito et al. [12] reported finding spot welds from 3 to 6 months after surgery, and the timing of their appearance was significantly earlier in proximal porous coating stems without an HA coating. In the present study, spot welds appeared even earlier than those previously reported. Oh et al. [13] showed the formation of spot welds at zone 6 after 6 months postoperatively with a DCPD-coated stem. However, the shape of the stem used in their study was different than that of the Excia, and earlier bone formation around the stem may have occurred in the present study.

Reactive lines were found in 30 hips (66%) at zone 4, and stress shielding tended to have advanced to, but not exceeded, the second degree. All of the stems were stable and not associated with proximal reactive lines, advanced subsidence, or pedestal formation. Pellegrini et al. [14] reported that radiolucent lines were seen around 30% of porous-coated stems, most commonly in zones 1, 4, and 7, and that these lines were not progressive. Ahmad Hatem et al. [15] followed up 138 patients using the Taperloc stem for a mean of 10 years and described that reactive lines were seen in the region of the stem in 75 hips (69%), most commonly in zone 4. Oh et al. [13] reported good clinical outcomes for a DCPD-coated stem, with reactive lines around the tip of the stem found in 35% of hips. Good mid-term and long-term results with the Bicontact stem (B. Braun, Melsungen, Germany) have been published [16–19]. Reactive lines were observed in 57 hips (66%) in the distal part after a mean follow-up of 2.8 years [16] and in 9 hips (8.7%) after a mean follow-up of 22.8 years [17]. Reactive lines in noncoated distal stem parts cannot be regarded as a sign of radiological loosening [15–17]. Like the Excia stem, the Bicontact stem has anteroposterior support flanges together with a lateral derotational wing, and the distal tapered part of the stem is smooth and uncoated. However, the Excia stem is shorter and more round-shaped in the distal part than the Bicontact stem. In mechanical studies on a stem with a proximal porous coating, the tensile stress was distributed on the lateral side of the distal portion of the stem and any large micromotion was found distally [20, 21]. Micromotion would also occur distally with the Excia stem because of differences in the coefficient of elasticity between the stem and the cortical bone when the load is not transferred through the proximal portion and the stem is stabilized at the middle portion. Proximal stress shielding also occurred and advanced to the second degree. Distal reactive lines tended to appear because the distal tip of the Excia stem is round-shaped and has a smooth surface.

**Figure 5:**
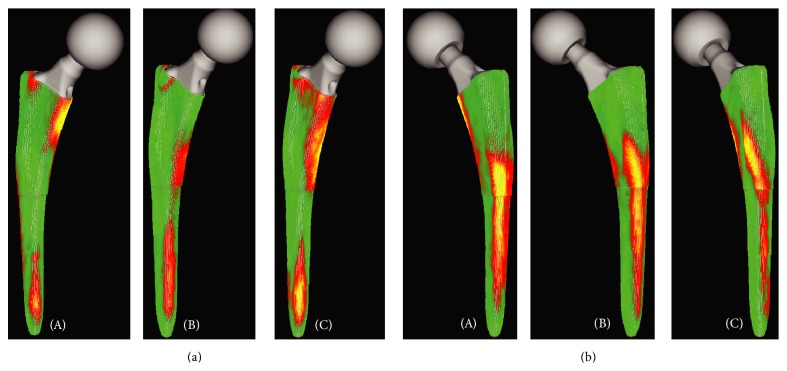
Posteromedial (a) and anterolateral (b) views of stems in contact with the cortical bone at the medial side of the proximal (A), middle (B), and both (C) portions.

Analysis of the CT images showed three contact patterns between the stem and the cortical bone, including contact in the proximal portion (Figure 5(A)), middle portion (Figure 5(B)), or both portions (Figure 5(C)) on the medial side of the stem. The stems were in contact with the bone at a high rate in the distal anterolateral (zone 3A) and posteromedial (zone 5P) portions (Figures 3(b) and 5). However, the portions in contact with the bone were not significantly correlated with any of the factors examined. Jingushi et al. [22] reported that the rate of secondary osteoarthritis caused by dysplastic hips was high in Japanese patients undergoing THA. Because dysplastic hip patients have narrow medullary canals [23] and high CFI scores, a correlation between the portions in contact with the bone and the CFI was expected. However, no such correlation was found, most likely because the fraction of normally shaped hips was quite high (91%, 31/34 hips) and the differences in the medullary canal shape had little effect.

Engh et al. [1] reported that distal fixation between the stem and the bone causes proximal stress shielding. Although the differences were not significant in the present study, the rate of contact at zone 7A tended to be lower and the appearance of spot welds at zones 2 and 6 tended to be higher in the second-degree stress shielding group. In other words, stress shielding tended to occur proximally if there was no contact at the medial portion proximally and spot welds were found at the middle portion (Figures 4(b) and 4(c)). Stress shielding did not always progress, even in cases where the stems were in contact with the bone at the distal portions (zones 3 and 5). However, distinct contact points were found at zone 2A in all hips, and the anterior flange was in contact with the cortical bone at the height of the lessor trochanteric tip (Figures 2(B) and 5).

Leali and Fetto [24] reported that loads were transferred through the proximal portion based on assessments of mean 4-year outcomes using lateral flare stems. A mechanical study by Arno et al. [25] indicated that proximal load transmission in cadaveric femurs implanted with lateral flare stems was similar to that in normally shaped femurs. Thus, the mid-lateral portion may contribute to the stability of the stem. Although the stem shape used in the present study was different, it is possible that the stability at the mid-lateral portion with the flange inhibited the progression of stress shielding beyond the second degree.

The validity of the evaluation method for detecting the portions of the stem in contact with the bone should be discussed, because measuring the cortical bone thickness in the proximal femur on CT images is difficult because it is very thin [26]. The thickness of the cortical bone at the neck of the femur is moderately different from that at the femur shaft. Therefore, we may be able to improve our technique by changing the threshold level slightly at the corticocancellous interface. In fact, we found instances where the CT values around the distal portion of the stem were more than 1200 HU and the stem was clearly mostly in contact at the distal portion. However, severe stress shielding of the proximal portion was not found in these cases (Figure 5(B)). Even if they were in contact distally, the stress transmission was also affected by differences in surface processing between the proximal porous coating and the distal smooth surface.

This study had several limitations. First, our analysis was retrospective, and the appearance of spot welds during the early period was not significant. Second, the number of hips was small and the fraction of normally shaped hips was quite high. In addition, there were no femurs with a stove-pipe canal in the present analysis using CT images. Sugano et al. [23] reported that the medullary canal torsion in dysplastic hips was different from that in normal hips. We speculated that the medullary canal torsion, stem alignments, and other femoral morphological factors influenced the contact patterns and clinical outcomes in hips with various morphological characteristics. Third, the follow-up periods were short. The reactive lines in zone 4 resulted in significantly higher stress shielding (second degree or greater) and the long-term results of the Excia stem were unclear. The reactive lines should be further examined to determine whether or not they progress during long-term follow-up. However, to the best of our knowledge, no previous studies have three-dimensionally analyzed the contact patterns between the stem and the cortical bone. The results reported here indicate that the stem was in contact with the bone at zone 2A in all cases. Further clinical studies of other stems are needed to validate this observed contact pattern.

## 5. Conclusions

THA using the Excia stem resulted in stable implants in the early postoperative phase. Using three-dimensional analysis of CT images, a distinct contact pattern was shown at zone 2A in all hips and the anterior flange was in contact with the cortical bone. However, the portions in contact with the bone and the presence of bone remodeling around the stem were not significantly correlated with stress shielding.
